# Malnutrition as the cause of recumbency in suckler cows associated with *Trypanosoma theileri* infection

**DOI:** 10.1186/s13028-020-00567-7

**Published:** 2021-01-09

**Authors:** Lilli Bittner, Kjelt Krämer, Adriana Wöckel, Teja Snedec, Cora Delling, Denny Böttcher, Gabor Köller, Walter Baumgartner, Wolfram Richardt, Alexander Starke

**Affiliations:** 1 Faculty of Veterinary Medicine, Clinic for Ruminants and Swine, An den Tierkliniken 11, 04103 Leipzig, Germany; 2 Faculty of Veterinary Medicine, Institute of Parasitology, An den Tierkliniken 35, 04103 Leipzig, Germany; 3 Faculty of Veterinary Medicine, Institute of Veterinary Pathology, An den Tierkliniken 33, 04103 Leipzig, Germany; 4 Tierarztpraxis FTA Dr. Gregor Stampa, Groß Floyen 8, 24616 Brokstedt, Germany; 5 University Clinic for Ruminants, University of Veterinary Medicine, Veterinärplatz 1, 1210 Vienna, Austria; 6 LKSmbH, August Bebel Str. 6, 09557 Lichtenwalde, Germany

**Keywords:** Blood parasites, Copper deficiency, Downer cow, Selenium deficiency

## Abstract

**Background:**

Recumbent cows are a diagnostic challenge because of a wide range of differential diagnoses, which include trauma, neurological and metabolic disorders, malnutrition and mineral deficiencies. This case report describes recumbent suckler cows that presented as a herd problem. In addition to weakness due to inanition, Cu and Se deficiencies were considered as possible aetiologies of the recumbency. Furthermore, *Trypanosoma (T.) theileri*, a blood parasite of unknown importance in Germany, was detected in the blood of some cows.

**Case presentation:**

Three recumbent cows were referred to the Clinic for Ruminants and Swine, Faculty of Veterinary Medicine of the University of Leipzig. They were unable to rise and had low body condition scores and rough hair coats. Haematological and serum biochemical analyses showed neutrophilia, electrolyte imbalances, increased activities of muscle and liver enzymes and decreased concentrations of trace elements, especially Copper (Cu) and Selenium (Se). *T. theileri* was detected in a routine blood smear from one cow. The cows did not respond to an intensive care protocol, which included intravenous fluids and electrolytes, mineral substitution, non-steroidal anti-inflammatories and antibiotics, and were therefore euthanized or died. Postmortem examination showed cachexia, subcutaneous and scleral oedema and muscular dystrophy, especially in the hind limbs. Follow-up examination of the herd of origin produced similar findings including the detection of *T. theileri* in a large proportion of the herd. Ration analysis revealed considerable undersupply of several nutrients.

**Conclusions:**

Based on all findings, an aetiological diagnosis of trace mineral and nutrient deficiency with possible involvement of *T. theileri* was made.

## Background

Recumbent cows that do not have hypocalcaemia, constitute a diagnostic challenge. The differential diagnoses include orthopaedic problems such as fractures, traumatic injuries of tendons and muscles and many other illnesses. High-producing dairy cows with hepatic dysfunction may present with hepatic encephalopathy [1] or hepatic coma. Infections causing cows to become recumbent include those affecting the central nervous system (e.g. listeriosis and meningitis) and peripheral nerves [1] as well as intoxications (e.g. botulism) [2]. End-stage systemic illness and prolonged energy and protein deficiencies (cachexia) may also cause cows to become recumbent [3]. Imbalances in the supply of macrominerals and trace elements, which are involved in many metabolic and enzymatic processes, usually cause nonspecific clinical signs in cattle but may also affect their ability to stand [4–6]. Nutritional muscular dystrophy (white muscle disease) [4] is caused by a deficiency of selenium (Se) and vitamin E and may result in cattle spending more time lying down. Copper (Cu) is another trace element that is associated with clinical signs affecting locomotion [7]. It is a co-factor for lysil oxidase, an enzyme involved in bone formation [8], and deficiency causes diminished strength of the bone [9]. The involvement of Cu deficiency in neuronal degeneration for cattle as in sheep is less described [10].

This case report describes a suckler cow herd with an increased incidence of recumbent cows. Malnutrition and trace element deficiencies were considered possible aetiologies for the clinical presentation. Furthermore, *T. theileri*, a blood parasite of unknown importance in German cattle, was detected some cows.

## Case presentation

### History

Three Fleckvieh cows were referred to the Clinic of Ruminants and Swine, University of Leipzig, in the winter of 2018 because they were unable to rise. The cows originated from the same year-round pasture-based suckler cow herd and were admitted within four days of each other. Five other recumbent cows from the same herd had died in the previous few weeks about a week after an initial favourable response to infusions of calcium, magnesium and glucose. The referred cows were 8, 9 and 10 years of age; one was about four months pregnant and the calves of the other two had been recently weaned. Two of the cows had been recumbent for a week but were still eating. Treatment on farm consisted of Se and vitamin E injections and calcium and glucose infusions. The third cow became recumbent on the day of referral. The tentative diagnosis made by the referring veterinarian was mineral deficiency.

### Clinical findings

All cows were examined clinically including neurological and orthopaedic assessment [11]. There were few obvious abnormalities. Salient points from the examination of each of the cows were as follows:

Cow no. 1 had a normal appetite, a poor body condition (body condition score [12] BCS 2.0) and a rough hair coat. The rectal temperature was 39.2 °C. The cow was able to move the limbs but could not rise. There was mild swelling of the right hind limb.

Cow no. 2, admitted at the same time as cow 1, had a poor body condition (BCS 2.0), a rough hair coat and a rectal temperature of 38.1 °C. The cow was unable to rise and the left hind limb appeared stiff but reacted to tactile stimulation.

**Fig. 1:**
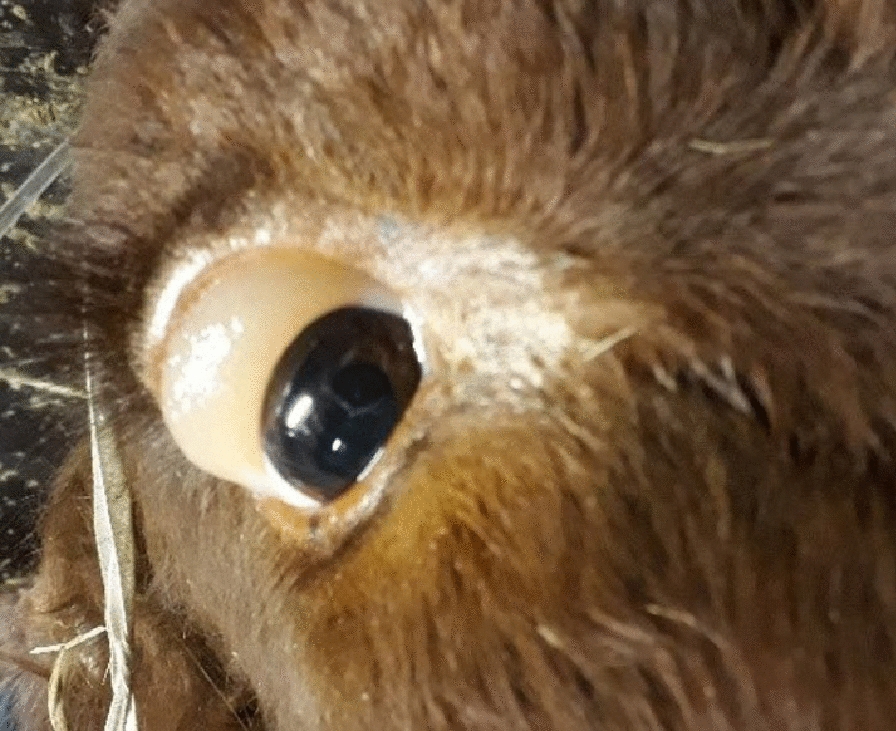
Severe scleral oedema affecting the right eye of cow 3 at the time of admission to our clinic

Cow no. 3 had a normal hair coat and a moderate body condition (BCS 3.0) but severely abnormal mentation and anorexia. The cow was somnolent and in lateral recumbency and the rectal temperature was 36.9 °C. Rumen fill was poor and there was severe scleral oedema in both eyes (Fig. 1).

Results of serum biochemical examination (jugular blood) are shown in Table 1. The concentrations of magnesium, potassium, chloride and albumin were below the reference intervals and the activities of aspartate aminotransferase (AST) were above the reference intervals in all cows. Two cows had calcium, sodium and total protein concentrations below the reference intervals and one cow had phosphorus, iron and bilirubin concentrations above the reference interval. Faecal tests showed mild to moderate infection with gastrointestinal nematodes. There was no ketonuria, and analysis of rumen fluid for botulism toxin was negative [13].

**Table 1:** Results of initial and final haematological and serum biochemical analyses in three cows referred to our clinic

Variable	Units	Reference interval [45]	Cow 1	Cow 1 Day 5	Cow 2	Cow 2 Day 16	Cow 3	Cow 3 Day 3
Haematocrit	L/L	0.24–0.46	0.29	0.27	0.22	0.20	0.26	0.29
Total leukocyte count	× 10^9^/L	5–10	5.0	3.2	5.1	15.1	5.1	2.4
Magnesium	mmol/L	0.9–1.32	0.43	0.67	0.56	0.41	0.80	0.55
Calcium	mmol/L	2–2.54	1.68	1.97	1.98	1.84	2.13	1.87
Phosphate	mmol/L	1.55–2.29	0.62	1.55	1.91	1.99	1.69	1.81
Potassium	mmol/L	3.9–5.2	2.52	5.22	2.93	4.78	2.71	3.85
Sodium	mmol/L	135–157	125	136	133	134	142	152
Chloride	mmol/L	95–110	93.3	88.0	89.1	98.0	83.9	95.0
Iron	µmol/L	13–33	18.0		6.3		14.0	
Total protein	g/L	68–82	61.8	73.0	67.5	72.6	78.4	73.0
Albumin	g/L	30–39	16.5	18.1	21.8	18.8	31.3	26.3
Bilirubin	µmol/L	3.3–5.3	2.9	5.5	3.8	3.8	3.4	2.6
Urea	mmol/L	2.0–6.8	5.8	6.3	5.6	8.9	6.3	13.7
Creatinine	µmol/L	55–150	84	45	86	59	64	98
GGT	U/L	< 50	18.9	54.6	22.0	77.9	34.5	52.5
AST	U/L	< 80	544.1	991.2	726.3	984.2	286.5	2931.7
CK	U/L	< 150	2580	2629	8505	1415	6951	42 494
GDLH	U/L	< 30	45.4	1070	9.1	1433	45.4	316.1

### Treatment

All cows received continuous intravenous (i.v.) infusion therapy during the hospitalisation including solutions of 0.9% NaCl (20–40 L), 40% glucose (3–12 L), potassium chloride (74.5 g/L, 100–200 mL) and calcium/magnesium (18.7 g calcium/L and 4.04 g magnesium/L, 0.5–1.5 L). The infusion rate of the different solutions was adjusted according to repeated blood testing. Additionally, all cows received Se and vitamin E (sodium selenite, alpha-tocopherol [Vitamin-E-Selen ad us. vet. 150 + 1.1 mg/mL] 4 mg/kg s.c.) and vitamin B complex (vitamin B_1_, B_2_, B_3_, B_12_, B_5_ and B_6_ [Vitamin-B-Komplex pro inj.] 15 mL s.c.) on the first or second day. All cows received meloxicam [Metacam^®^ 20 mg/mL], 0.5 mg/kg s.c. every other day. Cow 3 also received phosphorus and vitamin B12 on the first day ([Veyxol^®^ B-Phos], 100 ml per os.). The cows were moved to deep straw bedding, rolled from one side to the other twice or three times a day and closely monitored for feed and water intake. Because of a poor health status (cows 2 and 3) and co-morbidity (mastitis in cow 1), all cows were treated with sulfadimidine and trimethoprim [Trimethosel, 200 + 40 mg/mL], 26 mg/kg i.v.); cow 1 was treated for 3 days starting on day 6, cow 2 was treated for 7 days starting on day 6 and cow 3 was treated for 3 days starting on day 2. Cow 2 was switched to amoxicillin ([Betamox^®^ 150 mg/mL] 10 mg/kg i.m.) for 6 days because of a thrombophlebitis associated to the i.v. treatment and got a single dexamethasone injection ([Rapidexon Albrecht 2 mg/mL] 0.2 mg/kg i.v.) because of oedema in the lower neck region. It was a pitting oedema, which was not sensitive to palpation and had a normal surface temperature.

### Monitoring of laboratory variables

The results of Cu and Se analyses were available 3–5 days after admission and are shown in Table 2. During the period of hospitalisation, laboratory variables were measured four times in cow 1, nine times in cow 2 and three times in cow 3. In cows 1 and 2, the measurements temporarily shifted toward the reference intervals but were distinctly abnormal again toward the end of hospitalisation. In cow 3, laboratory variables did not normalise during hospitalisation. Values at the start and end of hospitalisation are shown in Table 1. In two cows, the haematocrit and the concentrations of magnesium, total protein and albumin were lower at the end of hospitalisation than at the start, whereas the concentrations of phosphorus, potassium and sodium increased in all cows. The chloride and calcium concentrations increased in one cow, and in another, the calcium and bilirubin concentrations and the activity of creatine kinase decreased below the initial values. The total white blood cell count decreased in two cows and increased in one. The activities of AST, gamma-glutamyl transferase (GGT) and glutamate dehydrogenase (GLDH) continually increased in all cows. The urea and creatinine concentrations increased in two cows.

**Table 2:** Serum levels of selenium and copper at the time of admission to our clinic in three cows

Variable	Units	Reference interval [45]	Cow 1	Cow 2	Cow 3
Selenium	µg/L	> 80	50.3	65.5	45.4
Copper	µmol/L	12.5–32.8	n.a	7.4	6.6

*n.a.* not analysed

### Outcome

At first, cows 1 and 2 continued to eat and drink during period of hospitalisation, and cow 2, but not cow 1, was able to rise with the help of a water tank (Aqua Cow Rise System- Denmark) and then using a harness. The health status of cow 1 deteriorated significantly when a phlegmonous mastitis developed five days after admission; and the cow was euthanised three days later because of complete anorexia.

**Fig. 2:**
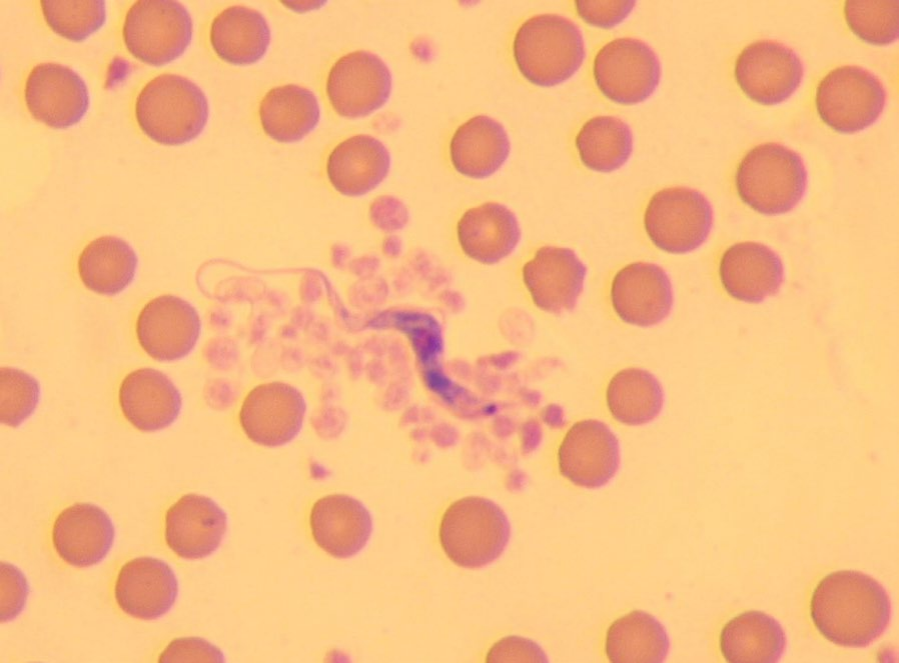
Extracellular blood parasite in a routine blood smear diagnosed as *Trypanosoma theileri* using PCR

Cow 2 developed ascites (detected via Ultrasonography (MyLabONE, Esaote) and abdominocentesis) and severe scleral oedema 14 days after admission. In routine haematological examination extracellular blood parasites were detected at this time (Fig. 2). The parasites was identified as *T. theileri* at the Institute of Parasitology and Tropical Veterinary Medicine, Free University of Berlin. The health status of the cow deteriorated and she was euthanized 22 days after admission.

Cow 3 did not respond to treatment and transfusion of 3 L of whole blood had no beneficial effect. The cow died spontaneously three days after admission.

### Pathological findings

In the gross examination all cows had cachexia and generalised subcutaneous oedema, which was accompanied by moderate (cows 1 and 2) or severe scleral oedema (cow 3). Other findings were purulent interdigital and palmar dermatitis combined with moderate to severe ichorous myositis of the right hind limb, and severe necrotising mastitis in cow 1, and pleural (approximately 2 L) and peritoneal effusion (approximately 300 mL) in cow 3. During histological examination in several muscle groups, particularly in the hind limbs, multiple necrotising foci were found. Stages of protozoan cysts consistent with *Sarcocystis* sp. were detected in various muscles. Parapoxvirus was detected in ulcerated areas of the oral mucosa (cows 1 and 3) and the mucosa of the rumen (cow 1) using polymerase chain reaction (PCR) (State Research Center for Health and Veterinary Sciences, Saxony). All cows had splenic haemosiderosis. In none of the examined organs parasitic stages of *T. theileri* were found during histological examination. In course of the hospitalisation, further diagnostics in the other cows supported the tentative diagnosis of a mineral deficency. Therefore in cow 3 a Cu analysis in the liver was performed. The Cu concentration was 8.1 µg/g dry liver (reference interval: 100–600 µg/g dry liver) [14].

### Description of herd of origin

The herd of origin was an EU-certified organic farm with 151 cattle (74 suckler cows, 2 bulls, 75 calves). Fertility was satisfactory but the owner reported poor growth rates in the calves born in the current year. This was currently a closed herd and all replacements were home-raised. The farm was pasture based and contained two pastures. The recumbent cows originated from one group of Fleckvieh cattle that were kept together on the same pasture. In the winter, part of the pasture was used during the daytime, but at night the cattle were housed in adjacent open barns and a partially covered yard. The pastures were sporadically fertilized with solid manure from the herd.

In the summer, the ration consisted primarily of grass and in the winter of hay, which was usually made on the farm but was purchased also from another source in the current year. Mineral blocks and loose minerals were offered ad libitum throughout the year, but it was not known how much the cattle consumed. There was no specific ration analysis but additional concentrate (wheat, rapeseed, molasses, minerals, vitamines) was fed after the episode with the recumbent cows. A macrocyclic lactone was used for parasite control.

A first farm visit was made in December 2018; the cows were fed hay *ad libitum* and water was available to groups of about 40 cattle from waterers with a ball closure. Many cattle were seen drinking from puddles of rain water. The cows were calm during the inspection. The BSC [12] varied widely among cows and ranged from 1.75 to 3.75 with an average score of 2.6. Likewise, there were very thin as well as well-nourished calves in the herd. Blood was collected from the coccygeal vessels of 27 cows for biochemical analysis. Healthy adult cows from the same group as the recumbent cows were selected. Because *T. theileri* had been identified in one of the referred cows, the cows were examined specifically for this blood parasite at the Institute of Parasitology, Leipzig University using conventional PCR (18SrDNA). Of ten PCR-positive samples, five were selected and analysed using Sanger deoxynucleotide sequencing (Microsynth Seqlab, Göttingen, Germany), and the diagnosis of *T. theileri* infection was confirmed. None of the cows had parasites detected in their blood smears.

Blood samples for biochemical analysis were collected again during the second farm visit four weeks later from 11 cows with different range of serum copper values in the first analyses that had been sampled in december. Laboratory variables outside the reference interval from both samples of these 11 cows are shown in Table 3. The same cows were retested for *T. theileri* and four were PCR-positive, but parasites were not detected in blood smears in any of them. Ten of the 11 cows from the second farm visit underwent liver biopsy for determination of liver Cu concentration (Table 3). This was achieved in standing cows under ultrasonographic guidance [15].

**Table 3:** Results of serum biochemical and liver biopsy analyses in 27 animals at the first farm visit and in 11 animals at the second farm visit

Variable	Units	Reference interval [45]	Visit 1 mean (min.–max.)	Visit 2 mean (min.–max.)
Magnesium	mmol/L	0.9–1.32	0.8 (0.6–1.1)	0.7 (0.6–0.9)
Potassium	mmol/L	3.9–5.2	5.6 (4.8–6.5)	5.3 (4.3–6.3)
AST	U/L	< 80	122 (95–169)	114 (94–143)
CK	U/L	< 150	251 (126–459)	160 (103–300)
Selenium (n = 8)	µg/L	> 80	43.1 (31.2–69.4)	77.9 (32.1–154.3)
Copper (n = 8)	µmol/L	12.5–32.8	9.1 (6.5–11.7)	13.9 (9–17.8)
Glutathione peroxidase	U/mL haematocrit	40–150		161 (68.3–220)
Copper in liver biopsy (n = 9)	µg/g dry liver	100–600		77.9 (32.1–154.3)

Blood was also collected from eight clinically healthy cows from another farm with comparable geographic location and housing management and tested for *T. theileri* using PCR, and four of the eight cows were positive.

Hay samples from two pastures used by the referred cows were analysed, and a ration was formulated based on the feed intake that had been established during history taking (Table 4).

**Table 4:** Results of analysis of hay samples from two different fields and the theoretical calculated ration with intake of 13.5 kg dry matter (DM) from hay sample 1 and 0.1 kg dry matter from the mineral supplement

		Hay sample 1	Hay sample 2	Mineral supplement	Calculated ration using hay sample 1
		In kg DM	In kg DM	In kg DM	Intake per animal
NEL	MJ/kg	4.6	5.7		62.1
Crude protein	G	52	88		702
Crude fibre	G	357	293		4819.5
Crude fat	G	15	15		202.5
Sugar	G	113	154		1525.5
Potassium	G	17.7	23.6		238.95
Calcium	G	3.2	3.4	110	54.2
Phosphate	G	2.2	2.5	60	35.7
Sodium	G	0.2	0.07	90	11.7
Magnesium	G	1.2	1.2	120	28.2
Chloride	G	2.8	3.6		37.8
Sulfur	G	1.2	1.4		16.2
Copper	Mg	3.6	4.5	1200	168.6
Zinc	Mg	17.7	20.8	6000	838.95
Manganese	Mg	103	104		1390.5
Iron	Mg	100	81		1350
Selenium	Mg	0.04	0.04	20	2.54
Molybdenum	Mg	1.59	0.36		21.465

### Recommendations and follow-up

We strongly recommended improving the ration and adding more waterers to the barn and pastures. The owner was advised to administer a mineral bolus (Rumifert^®^), which releases 78 mg Cu, 2.99 mg cobalt (Co) and 1.7 mg Se per day for 4–6 months (Rumifert^®^) to adult cows with a low BCS. The owner agreed to administer the bolus to thin cows but did not agree with feeding additional concentrate during the summer. Until present (December 2020), there were no new cases of recumbent cows and the owner thought the weight gains of the calves were acceptable.

## Discussion and conclusions

All three cows referred to our clinic were recumbent cows, had a poor BCS and a rough hair coat. Additionally, one of the cows had a severely abnormal demeanour and severe scleral oedema. Serum biochemical testing in all cows showed electrolyte imbalances, increased activities of enzymes and decreased concentrations of Se and Cu. The biochemical findings in the cows tested during the farm visits mirrored those of the cows referred to the clinic, and the same was true for the detection of *T. theileri*. The farm visits made it clear that the underlying problems in this herd were inadequate nutrition. This led to a tentative diagnosis of recumbency in cows attributable to chronic energy, protein, Se and Cu deficiencies potentially associated with *T. theileri* infection.

Poor body condition and rough hair coat have been associated with trace element deficiencies [5, 16, 17] but lack of protein and energy in the ration can have similar clinical manifestations [18]. Subcutaneous oedemas seen in all cows and the pronounced scleral oedema in cow 3 were possibly related to cachexia, which is accompanied by hypoalbuminaemia causing an osmotic oedema. Albumin maintains the colloid osmotic pressure in the blood plasma thus preventing the escape of water into the interstitial space. Malnutrition and in turn protein and trace element deficiencies limit albumin synthesis in the liver. Whether an infection with *T. theileri* accelerated or worsened an oedema formation cannot be clarified in our case.

The results of blood and liver analysis in the referred cows and the cows examined on farm suggested Se and Cu deficiencies. The concentrations in blood reflect the current trace element supply in the ration, whereas the Cu concentrations in liver tissue and the serum activity of glutathione peroxidase reflect the supply of these elements over a longer period of time. The mean Cu concentration of 77.9 µg/g dry liver was suggestive of a prolonged deficiency. However, published reference intervals for trace elements vary considerably. For the Se concentration in serum we used > 80 µg/L as the cut-off compared with > 40 µg/L in an earlier study [19]. If the latter cut-off had been used, the Se concentration of most of the cows tested in the present study would have been in the reference interval. Likewise, the cut-off concentration of Cu of > 100 µg/g dry liver [14] used in this study was considerably higher than > 33 µg/g dry liver published in another source [20]. However, two cows had Cu concentrations < 33 µg/g dry liver confirming that the herd suffered from Cu deficiency.

Other trace element deficiencies, for instance those caused by a lack of cobalt (Co), cause non-specific clinical signs including weight loss, rough hair coat and anaemia [21]. The Co concentration was not analysed for cost reasons but in view of the undersupply of Se and Cu it is likely that Co was deficient too.

Trace element deficiencies may be primary or secondary; primary implies undersupply in the ration and secondary means that the amount in the ration is sufficient but a deficiency is caused by impaired absorption or metabolism associated with another trace element. Primary Se and Cu deficiencies typically occur when the soil has low Se and Cu levels and cattle graze or are fed hay harvested from these fields. Additional trace elements were not applied to the farm fields described in this study because only manure from the cows was used. Soil maps provided by the “Sächsische Landesamt für Umwelt, Landwirtschaft und Geologie” showed that the soil of the farms area is relatively low in Se, and targeted fertilizing of fields with Se is therefore indicated when a deficiency in the soil is confirmed. The same is true for Cu. Analysis of the hay revealed low Se and Cu levels, but it is not clear whether these deficiencies could have been corrected by ad libitum mineral feeding. However, the low serum concentrations suggest that they were not corrected. With a secondary deficiency, Cu may form complexes with sulphur (Su) or molybdenum (Mo) that reduce Cu absorption or its availability in the blood [17]. The Cu to Mo ratio in the ration should be smaller than 2:1 [22].

The protein and energy contents of the ration were also problematic. Two hay samples had 52 and 88 g crude protein/kg dry matter, which was considered very low. The Committee for Requirement Standards of the Society of Nutrition Physiology (GfE) recommends a ratio of greater than 12:1 for crude protein (g): metabolic energy (MJ) to ensure adequate microbial protein synthesis and rumen function [23]. This ratio was 6:1 for sample 1 and 9:1 for sample 2. A lack of nitrogen limits the microbial protein synthesis and thus the availability of essential amino acids for the protein synthesis [24].The herd problem described in this report shows that feeding poor-quality hay as the sole ration outside of the pasture season does not meet the nutritional requirements of cows and leads to deficiencies in protein, energy, macrominerals and trace elements. Supplemental feeding of concentrate and mineral was started shortly before the first farm visit and a significant improvement in the serum concentration of trace elements at the herd level was evident as early two months later at the second visit.

In addition to nutritional problems also infectious agents might have played a role in the clinical presentation. Parapoxvirus causes bovine stomatitis, which is usually a self-limiting, mild viral disease [25]. It is unlikely that this virus played a major role in the cases described in this report. The significance of *T. theileri* is less clear. This is a flagellate protozoan blood parasite with trypomastigote and epimastigote forms that can be found also in lymph and cerebrospinal fluids [26]. Transmission is via bloodsucking insects and arachnarids; horse flies (*Tabanidae*) [27] and hard ticks (*Ixodidae*) are important vectors in cattle. The vectors are accidentally consumed with feed and crushed during chewing, which releases the parasites allowing them to invade the host through the oral mucosa [28]. Infection with *T. theileri* usually does not cause clinical signs [27, 29, 30] and in most cases is an incidental finding. However, there have been occasional reports of infected cattle with fever, anaemia, petechia of the mucous membranes, tachycardia and tachypnoea [31–33] and sporadic reports of peritonitis [34], arthritis [35] and meningoencephalitis [36]. In many parts of the world, *T. theileri* has a prevalence of up to 80% in the cattle population and in the European Union the parasite has been reported in many countries, including Germany [27, 28, 32, 37–39]. It is assumed that this parasite causes clinical illness in immunocompromised patients and those with co-morbidities [35]. Cow 2 had anaemia at the end of hospitalisation but whether this was due to *T. theileri* [31–33] is questionable considering the wide range of other problems. PCR-positive cows did not have anaemia and the results of cows that were tested twice were not consistently positive, similar to a previous report [31]. Furthermore, another herd of clinically healthy cows that was tested for control purposes had *T. theileri* PCR-positive cows. Therefore, the aetiological significance of this parasite for recumbent cows remains unclear. The fact that the parasites were detected in blood smears suggests that the parasite load in the referred cow was high, whereas blood smears were negative in the PCR-positive cows tested on-farm. The lower sensitivity of blood smears compared with PCR for the diagnosis of trypanosomiasis has been reported previously [40, 41]. Cattle with subclinical or chronic infection generally have very mild parasitaemia [40–42] that can only be diagnosed using PCR.

In view of global warming, it would seem advisable to study vector-borne pathogens because of the likely expansion of the range of vectors. An example is the spread of bluetongue virus that has occurred in recent years [43]. To further clarify the involvement of *T. theileri* in the aetiology of poor-doing cows, the prevalence of this parasite in suckler herds and its clinical manifestation should be investigated.

In summary, the problem of recumbency in suckler cows described in this report appears to have had a multifactorial aetiology. The cows had clinical and pathological findings that were consistent with energy, protein, mineral and trace element deficiencies. These findings were mirrored at the herd level by heterogeneous body condition scores and low trace element concentrations in blood and liver samples. The importance of adequate nutrition should be stressed particularly in extensive beef production systems that are based on minimal labour and fertilizer inputs. These minimal inputs can cause animal welfare problems. Organic feed and natural life itself are not enough to guarantee a good quality of life. Therefore, intensive monitoring and agricultural knowledge on the farmer’s side is necessary to sustain healthy animals [44].

The relevance of *T. theileri* in our case is not clear, however could have worsened the clinical course and therefore need further investigation.

## Data Availability

All data generated or analysed during this study are included in this article.

## Prior publication

Data have not been published previously.
